# Supplementary material for: Optimization of Ultrasound-Assisted Extraction of Bioactive Compounds from Pelvetia canaliculata to Sunflower Oil
Source: Foods. 2021 Jul 27;10(8):1732. doi: 10.3390/foods10081732 (PMC8391403; doi:10.3390/foods10081732)
Supplement: Supplementary file 1 [file foods-10-01732-s001.zip › foods-1286577-supplementary.pdf]

**Supplementary Material**

## S.1 Determination of chromatic characteristics

The colour of the samples was analysed according to the Portuguese Standard NP 937 from 1972. UV-Vis spectrophotometer (Agilent Technologies cary series 100 UV-Vis) was used for determination of the transmittance of the oil samples, in cells with 5 mm optical path, at wavelengths 445 nm, 495 nm, 560 nm, 595 nm and 625 nm, using carbon tetrachloride as reference. Three replicates of each sample were analysed. The values of transmittance were used to calculate the following attributes: chromatic coordinates $(x, y)$, transparency $(Y)$, dominant wavelength $(\lambda)$ and purity $(\sigma)$. Chromatic coordinates correspond to the abscissa $x$ and ordinate $y$ of the CIE (Commission Internationale d'Eclairage) chromaticity diagram corresponding to the light transmitted by the oil. The transparency is defined as percentage ($Y$%) of incident light, transmitted after passing through the oil phase; the dominant wavelength is defined as spectral radiation λ, expressed in nanometres (nm), which predominates in the light transmitted by the oil and the purity is defined as percentage (σ %) of the light with the dominant wavelength, in the beam of light transmitted by the oil.

The chromatic coordinates, transparency, dominant wavelength, and purity of the original oil and of the supplemented oils, are presented in Table S1.

Non-supplemented oil sample (13) has chromatic coordinates, x and y, near the illuminant C point in the chromaticity diagram of the CIE system. All the supplemented oils show higher x and y values indicating a colour change, in the yellow region around the illuminant A, due to pigment extraction from the algae to the oils.

The transparency of supplemented oils varied from 19.9 to 45.2 %. These values are much lower than the value of the original refined sunflower oil (sample 13: 96.9 %), which can result in problems of sensory acceptance by the consumers. Statistical analysis of the CCRD results showed that only the extraction time has a quadratic negative effect on the transparency of the oils. Therefore, the effect of algae concentration on this parameter was not significant and no response surface could be fitted to the experimental results.

The dominant wavelength was constant and equal to 577 nm in all the supplemented oils, which shows to be independent from the algae concentration or the UAE time used in each experiment.

**Table S1:** CCRD results for the chromatic properties of each sample of supplemented oil, for the original sunflower oil. Sample 13 is the original oil with a 20 min ultrasound treatment.

| | | | **Chromatic features** | | | | | |
|---|---|---|---|---|---|---|---|---|
| **Assay** | **Algae (% m/v)** | **UAE time (min)** | **Chromatic coordinates** | | **Transparency** | **Dominant wavelength (nm)** | **Purity** |
| | | | **x** | **y** | **Y %** | **λ** | **σ (%)** |
| 1 | 7.2 | 7.2 | 0.415 | 0.427 | 28.6 | 577 | 57.9 |
| 2 | 7.2 | 17.8 | 0.431 | 0.44 | 22.5 | 577 | 65.6 |
| 3 | 17.8 | 7.2 | 0.459 | 0.47 | 22.5 | 577 | 81.0 |
| 4 | 17.8 | 17.8 | 0.456 | 0.465 | 22.6 | 577 | 78.8 |
| 5 | 5.0 | 12.5 | 0.392 | 0.405 | 45.0 | 577 | 45.7 |
| 6 | 20.0 | 12.5 | 0.451 | 0.467 | 37.3 | 577 | 77.9 |
| 7 | 12.5 | 5.0 | 0.458 | 0.468 | 20.3 | 577 | 80.1 |
| 8 | 12.5 | 20.0 | 0.448 | 0.457 | 19.9 | 577 | 74.5 |
| 9 | 12.5 | 12.5 | 0.445 | 0.458 | 30.9 | 577 | 73.9 |
| 10 | 12.5 | 12.5 | 0.437 | 0.451 | 36.5 | 577 | 70.1 |
| 11 | 12.5 | 12.5 | 0.45 | 0.461 | 27.5 | 577 | 76.1 |
| 12 | 12.5 | 12.5 | 0.449 | 0.461 | 45.2 | 577 | 76.0 |
| 13 | 0.0 | 20.0 | 0.317 | 0.327 | 96.9 | 572 | 4.5 |

The colour purity of supplemented oils varied between 45.7 and 81.0 %, while the value of the original refined sunflower oil was 4.5 %. Statistical analysis of the colour purity of samples showed that algae concentration had a significant positive effect (p= 0.03) on this parameter. In addition, the quadratic positive effect of extraction time cannot be ignored. Thus, a response surface (Fig. S1) described by the following equation can be fitted to the experimental results ($R^2$=0.613; $R^2_{Adj}$ = 0.527):

$$Purity(\%) = 43.795 + 1.931 \times [algae] + 0.006t^2 \qquad (S1)$$

where [algae] is the concentration of the algae, expressed in % (m/v) and t corresponds to ultrasound extraction time (min).

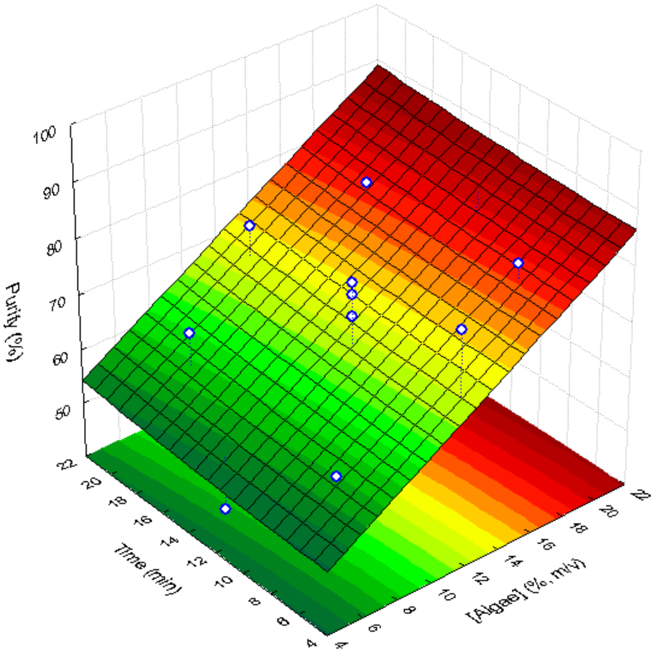


**Figure S1:** Response surface describing the effect of algae concentration and UAE time on colour purity of the supplemented oils.

This response surface, describing the colour purity of supplemented samples (Figure S1), shows similar dependence on algae concentration and UAE time as chlorophyll and carotenoid contents. In fact, the purity showed to increase with chlorophyll and carotenoid contents in the oils, following logarithmic models (Fig. S2).

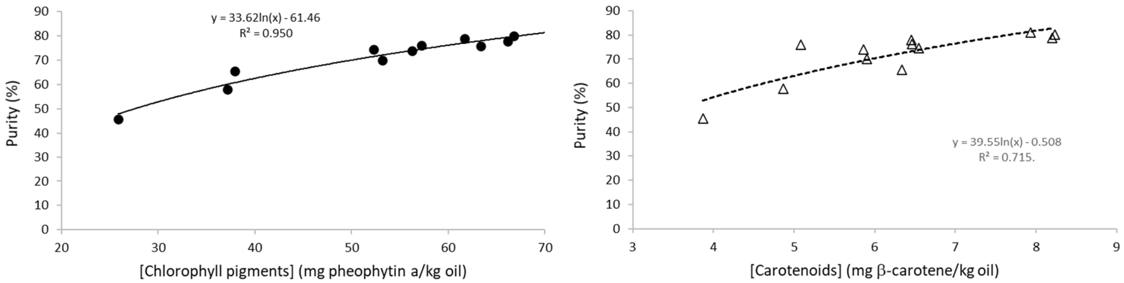

**Figure S2:** Relationships between colour purity of supplemented oil samples and concentration of chlorophylls and carotenoids.
